# External Periostitis

**Published:** 1866-06

**Authors:** Henry S. Chase

**Affiliations:** Iowa City, Iowa


### EXTERNAL PERIOSTITIS.

BY HENRY S. CHASE, D. D. S., IOWA CITY, IOWA.

This is an affection located on the gingival portion of the alveolo-dental periostuem. It is that part which surrounds the neck of the tooth, and usually covered with the gum, a little below its free margin.

DIAGNOSIS.—Parts mentioned, very sensitive to the touch, also to cold air or cold water. The extent of the sensitive portion does not exceed a line in width. *Decay*, situated at this portion of the tooth, will produce similar symptoms; but in this disease there is no decay, the parts are not disintegrated in the least. The prognosis is favorable, render proper treatment. Without treatment it continues for weeks and months, and many cases that have come to me have requested extraction. But this is unnecessary—a cure is certain in a few days under the following treatment: *Paint*, with a slender pointed quill tooth-pick, the inflamed part with pure creosote, every morning, or once a day until cured. Three or four applications will seldom fail to cure, and sometimes a single one will do.

This is a very annoying complaint, and one which I do not remember seeing "in the books." This short paper on the subject was suggested by the enquiries of a brother practitioner, not knowing how to cure the disease, and being surprised when informed of the simple remedy. Perhaps some others may be benefitted by reading *this*.

